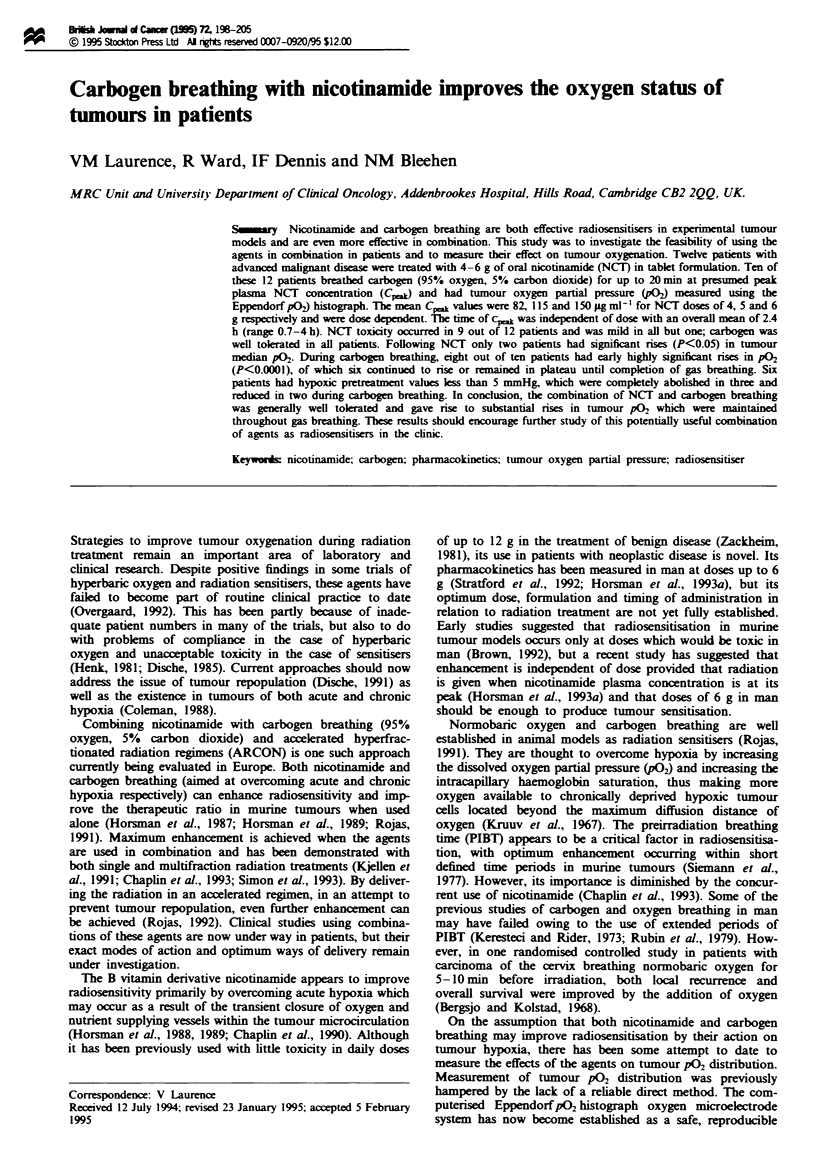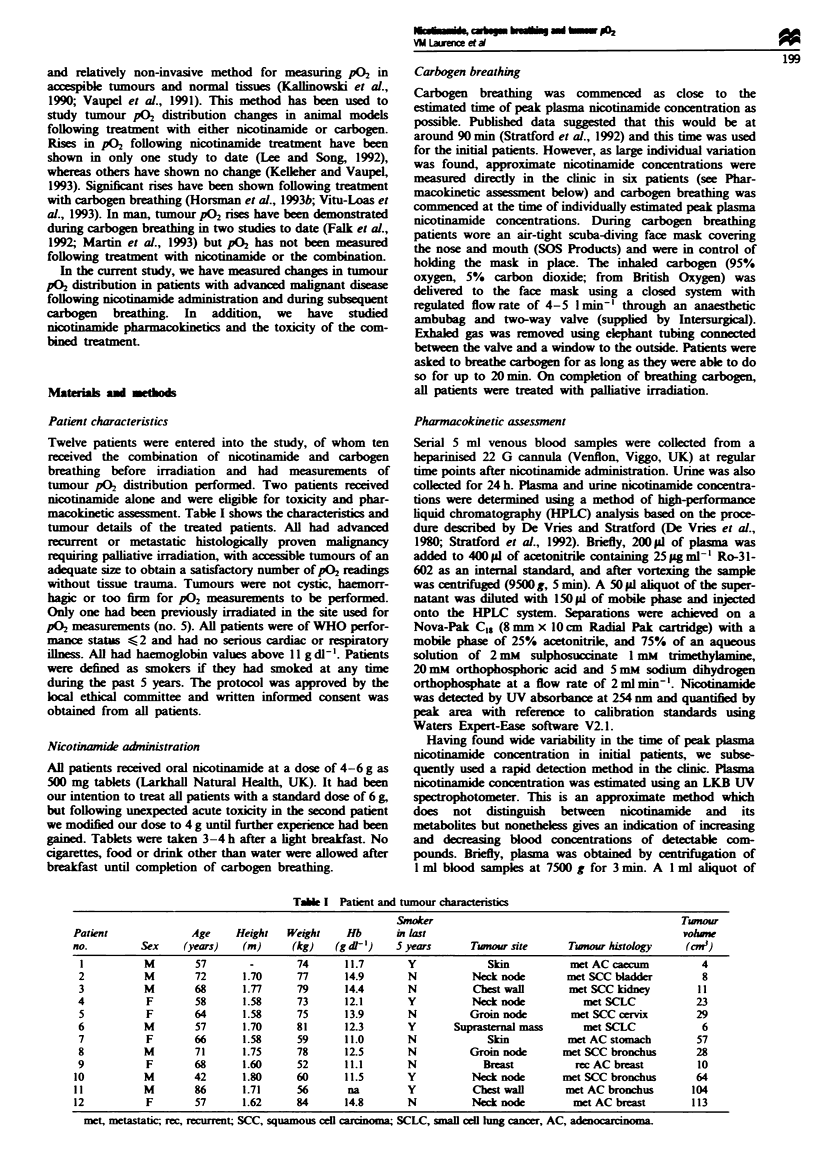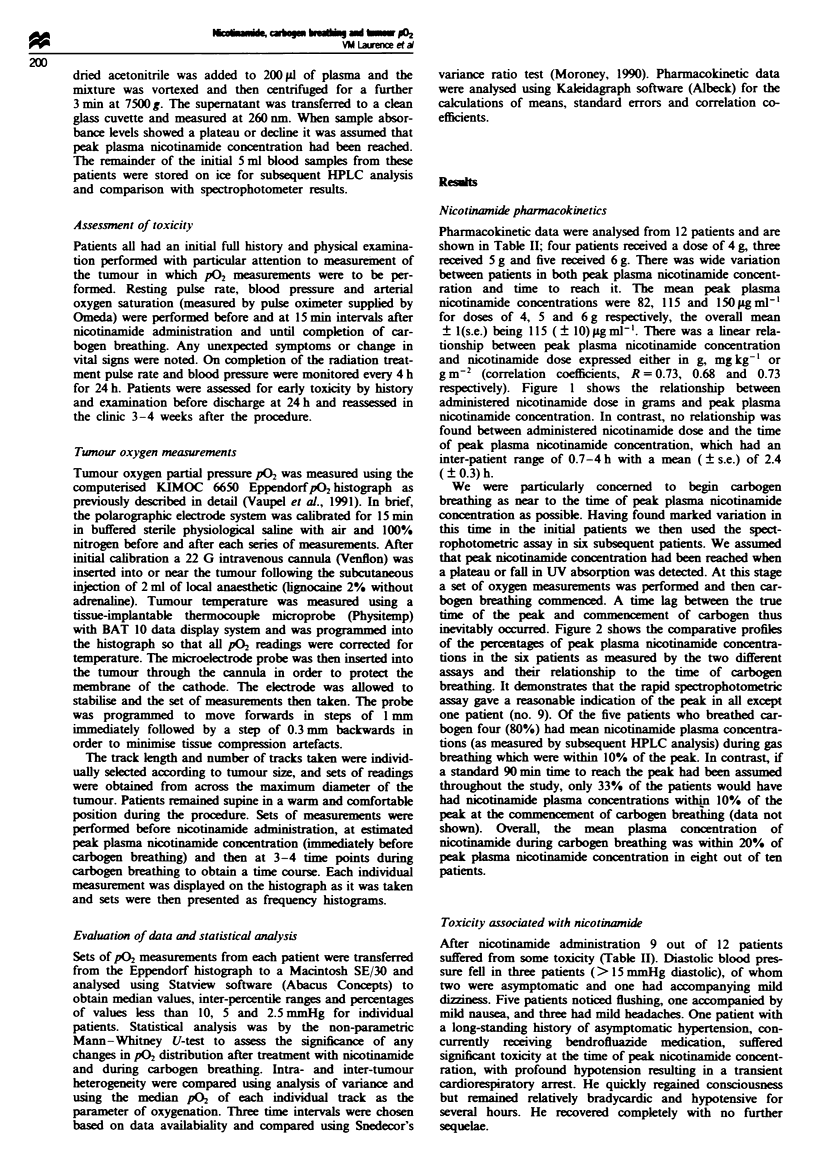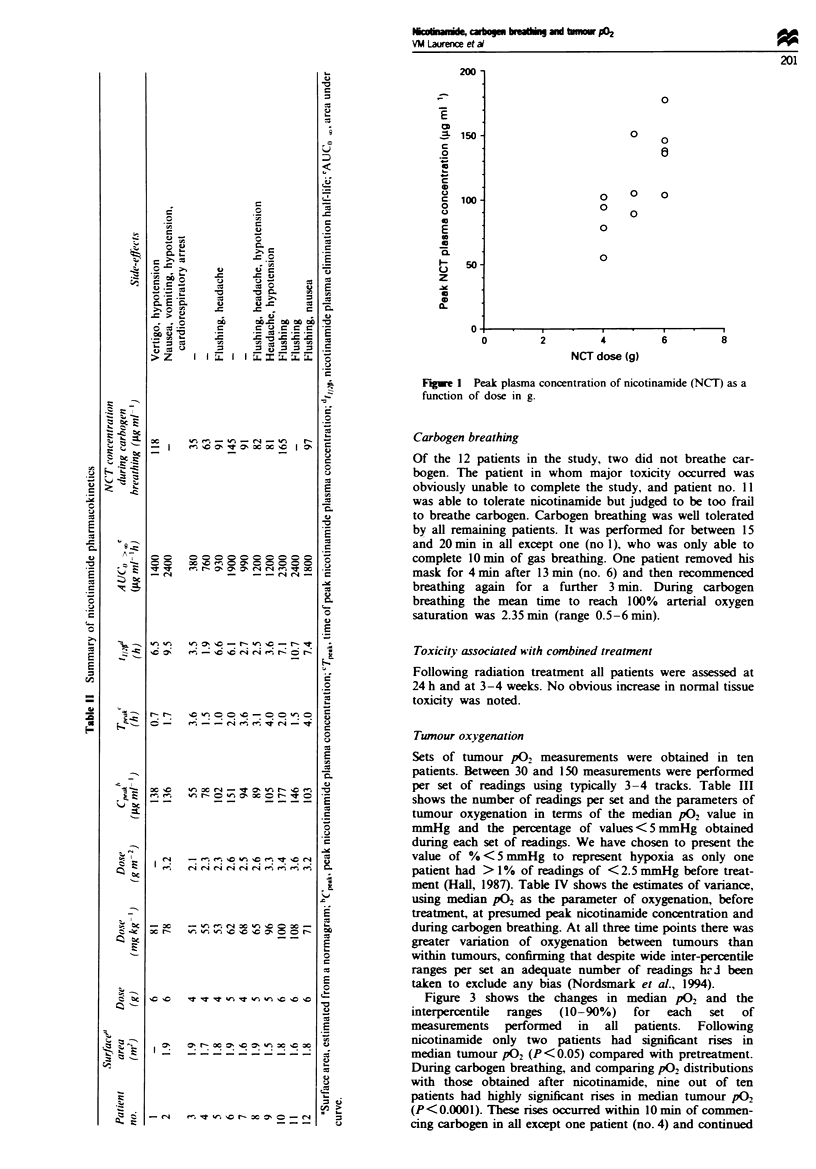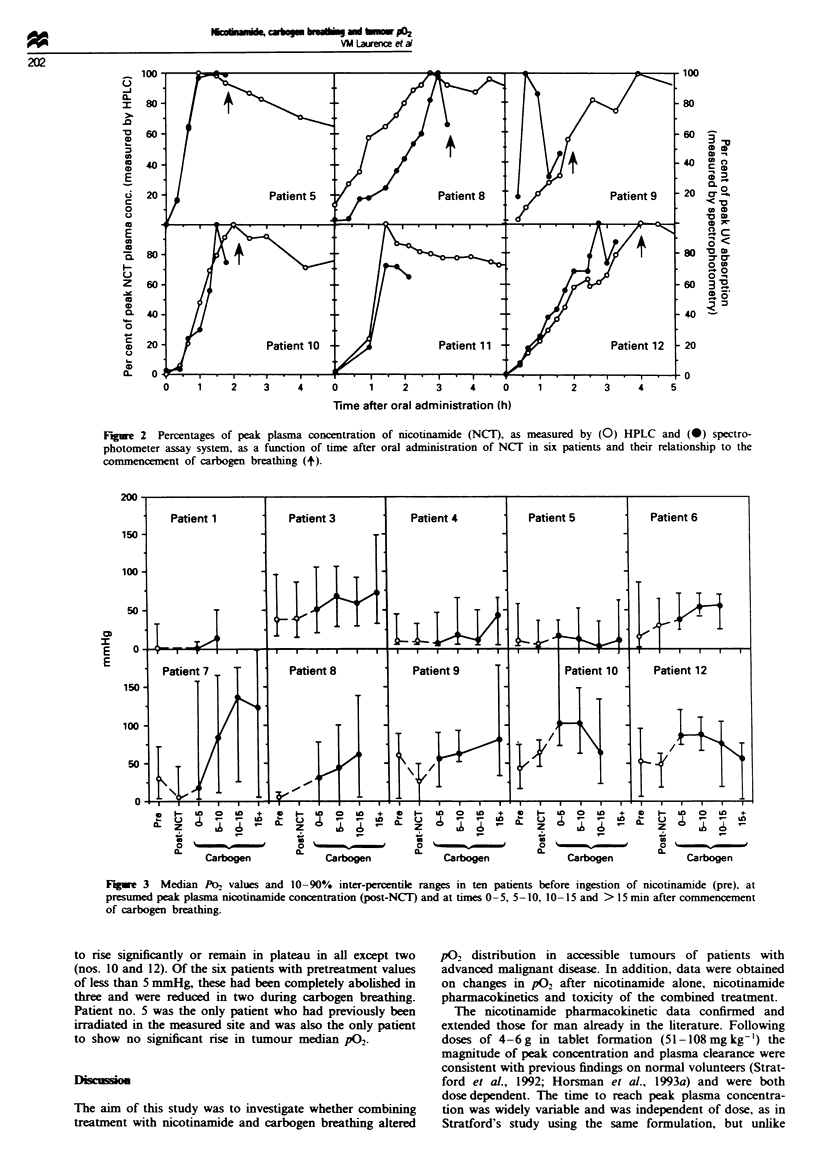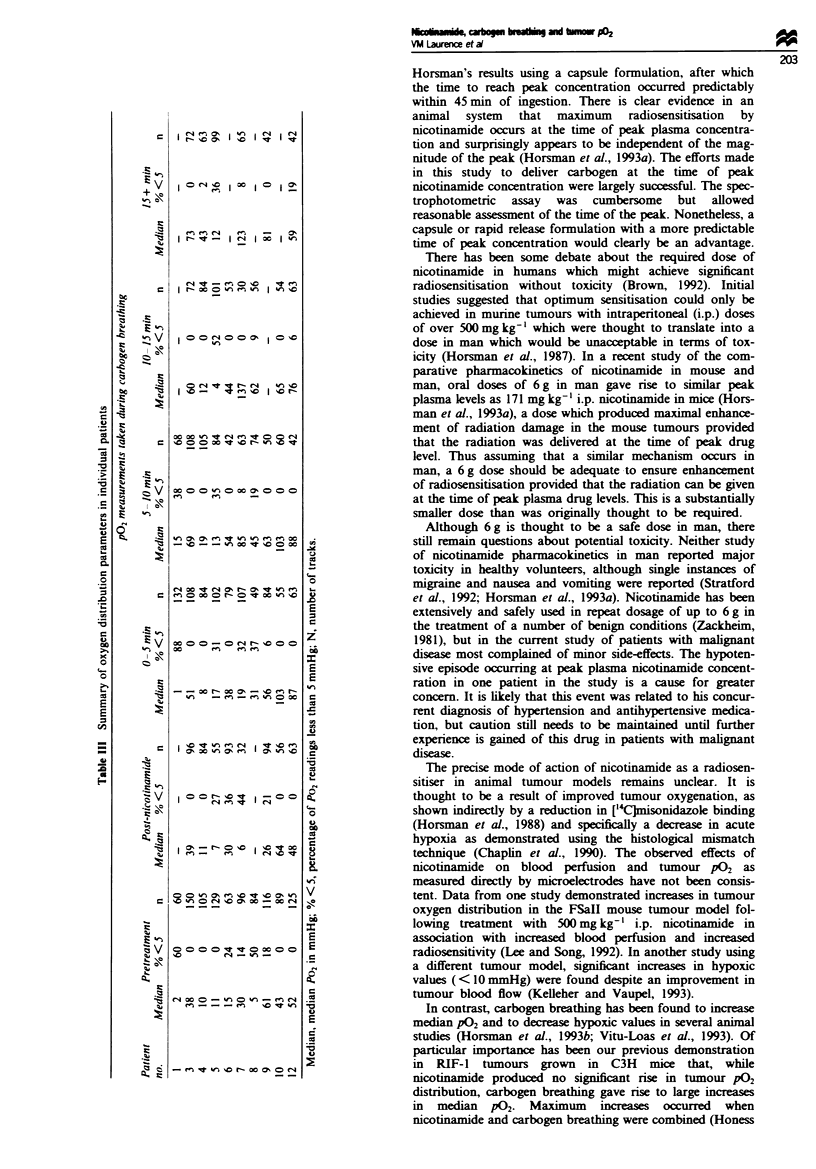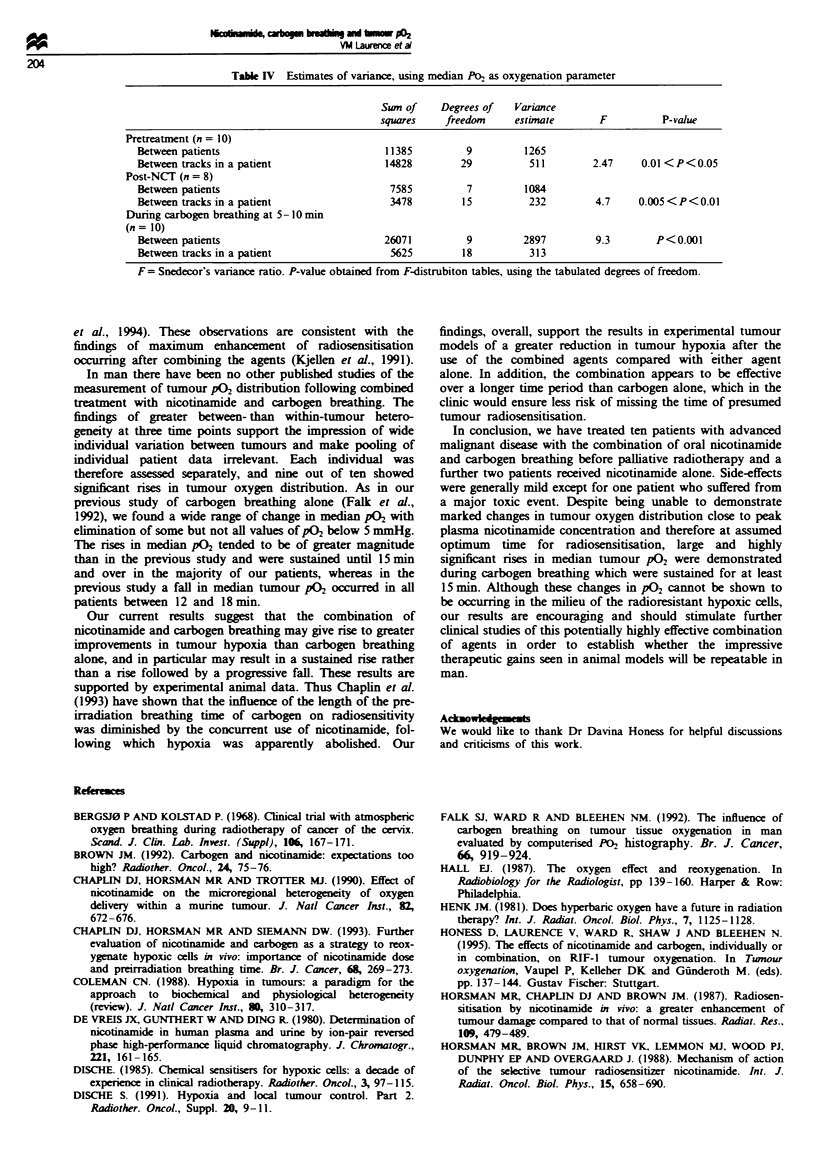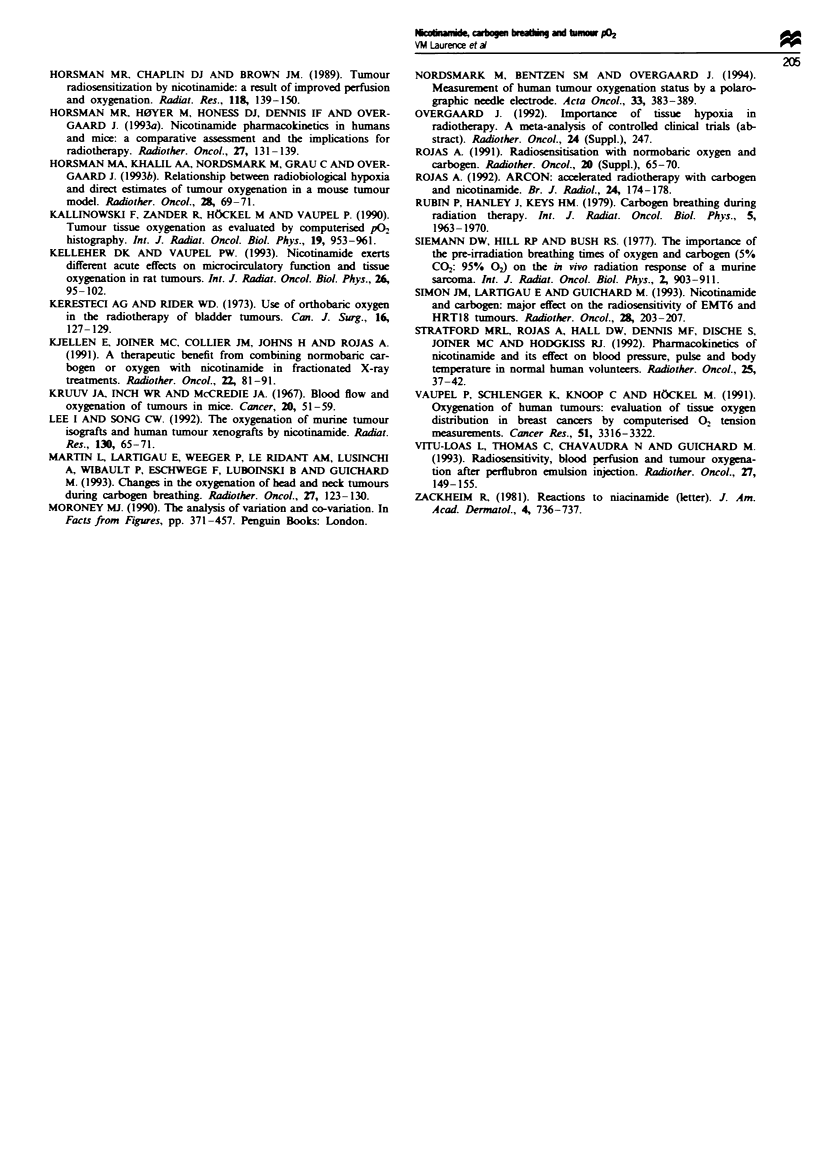# Carbogen breathing with nicotinamide improves the oxygen status of tumours in patients.

**DOI:** 10.1038/bjc.1995.303

**Published:** 1995-07

**Authors:** V. M. Laurence, R. Ward, I. F. Dennis, N. M. Bleehen

**Affiliations:** MRC Unit, Addenbrookes Hospital, Cambridge, UK.

## Abstract

Nicotinamide and carbogen breathing are both effective radiosensitisers in experimental tumour models and are even more effective in combination. This study was to investigate the feasibility of using the agents in combination in patients and to measure their effect on tumour oxygenation. Twelve patients with advanced malignant disease were treated with 4-6 g of oral nicotinamide (NCT) in tablet formulation. Ten of these 12 patients breathed carbogen (95% oxygen, 5% carbon dioxide) for up to 20 min at presumed peak plasma NCT concentration (Cpeak) and had tumour oxygen partial pressure (pO2) measured using the Eppendorf pO2) histograph. The mean Cpeak values were 82, 115 and 150 micrograms ml-1 for NCT doses of 4, 5 and 6 g respectively and were dose dependent. The time of Cpeak was independent of dose with an overall mean of 2.4 h (range 0.7-4 h). NCT toxicity occurred in 9 out of 12 patients and was mild in all but one; carbogen was well tolerated in all patients. Following NCT only two patients had significant rises (P < 0.05) in tumour median pO2. During carbogen breathing, eight out of ten patients had early highly significant rises in pO2 (P < 0.0001), of which six continued to rise or remained in plateau until completion of gas breathing. Six patients had hypoxic pretreatment values less than 5 mmHg, which were completely abolished in three and reduced in two during carbogen breathing. In conclusion, the combination of NCT and carbogen breathing was generally well tolerated and gave rise to substantial rises in tumour pO2 which were maintained throughout gas breathing. These results should encourage further study of this potentially useful combination of agents as radiosensitisers in the clinic.


© 1995 Stockton Press Ltd All rights reserved 0007-0920/95 $12.00

### Carbogen breathing with nicotinamide improves the oxygen status of tumours in patients

VM Laurence, R Ward, IF Dennis and NM Bleehen

*MRC Unit and University Department of Clinical Oncology, Addenbrookes Hospital, Hills Road, Cambridge CB2 2QQ, UK.*

**Summary** Nicotinamide and carbogen breathing are both effective radiosensitisers in experimental tumour models and are even more effective in combination. This study was to investigate the feasibility of using the agents in combination in patients and to measure their effect on tumour oxygenation. Twelve patients with advanced malignant disease were treated with 4–6 g of oral nicotinamide (NCT) in tablet formulation. Ten of these 12 patients breathed carbogen (95% oxygen, 5% carbon dioxide) for up to 20 min at presumed peak plasma NCT concentration ($C_{peak}$) and had tumour oxygen partial pressure ($pO_2$) measured using the Eppendorf $pO_2$) histograph. The mean $C_{peak}$ values were 82, 115 and 150 $\mu g\ ml^{-1}$ for NCT doses of 4, 5 and 6 g respectively and were dose dependent. The time of $C_{peak}$ was independent of dose with an overall mean of 2.4 h (range 0.7–4 h). NCT toxicity occurred in 9 out of 12 patients and was mild in all but one; carbogen was well tolerated in all patients. Following NCT only two patients had significant rises ($P$<0.05) in tumour median $pO_2$. During carbogen breathing, eight out of ten patients had early highly significant rises in $pO_2$ ($P$<0.0001), of which six continued to rise or remained in plateau until completion of gas breathing. Six patients had hypoxic pretreatment values less than 5 mmHg, which were completely abolished in three and reduced in two during carbogen breathing. In conclusion, the combination of NCT and carbogen breathing was generally well tolerated and gave rise to substantial rises in tumour $pO_2$ which were maintained throughout gas breathing. These results should encourage further study of this potentially useful combination of agents as radiosensitisers in the clinic.

**Keywords:** nicotinamide; carbogen; pharmacokinetics; tumour oxygen partial pressure; radiosensitiser

Strategies to improve tumour oxygenation during radiation treatment remain an important area of laboratory and clinical research. Despite positive findings in some trials of hyperbaric oxygen and radiation sensitisers, these agents have failed to become part of routine clinical practice to date (Overgaard, 1992). This has been partly because of inadequate patient numbers in many of the trials, but also to do with problems of compliance in the case of hyperbaric oxygen and unacceptable toxicity in the case of sensitisers (Henk, 1981; Dische, 1985). Current approaches should now address the issue of tumour repopulation (Dische, 1991) as well as the existence in tumours of both acute and chronic hypoxia (Coleman, 1988).

Combining nicotinamide with carbogen breathing (95% oxygen, 5% carbon dioxide) and accelerated hyperfractionated radiation regimens (ARCON) is one such approach currently being evaluated in Europe. Both nicotinamide and carbogen breathing (aimed at overcoming acute and chronic hypoxia respectively) can enhance radiosensitivity and improve the therapeutic ratio in murine tumours when used alone (Horsman *et al.*, 1987; Horsman *et al.*, 1989; Rojas, 1991). Maximum enhancement is achieved when the agents are used in combination and has been demonstrated with both single and multifraction radiation treatments (Kjellen *et al.*, 1991; Chaplin *et al.*, 1993; Simon *et al.*, 1993). By delivering the radiation in an accelerated regimen, in an attempt to prevent tumour repopulation, even further enhancement can be achieved (Rojas, 1992). Clinical studies using combinations of these agents are now under way in patients, but their exact modes of action and optimum ways of delivery remain under investigation.

The B vitamin derivative nicotinamide appears to improve radiosensitivity primarily by overcoming acute hypoxia which may occur as a result of the transient closure of oxygen and nutrient supplying vessels within the tumour microcirculation (Horsman *et al.*, 1988, 1989; Chaplin *et al.*, 1990). Although it has been previously used with little toxicity in daily doses of up to 12 g in the treatment of benign disease (Zackheim, 1981), its use in patients with neoplastic disease is novel. Its pharmacokinetics has been measured in man at doses up to 6 g (Stratford *et al.*, 1992; Horsman *et al.*, 1993*a*), but its optimum dose, formulation and timing of administration in relation to radiation treatment are not yet fully established. Early studies suggested that radiosensitisation in murine tumour models occurs only at doses which would be toxic in man (Brown, 1992), but a recent study has suggested that enhancement is independent of dose provided that radiation is given when nicotinamide plasma concentration is at its peak (Horsman *et al.*, 1993*a*) and that doses of 6 g in man should be enough to produce tumour sensitisation.

Normobaric oxygen and carbogen breathing are well established in animal models as radiation sensitisers (Rojas, 1991). They are thought to overcome hypoxia by increasing the dissolved oxygen partial pressure ($pO_2$) and increasing the intracapillary haemoglobin saturation, thus making more oxygen available to chronically deprived hypoxic tumour cells located beyond the maximum diffusion distance of oxygen (Kruuv *et al.*, 1967). The preirradiation breathing time (PIBT) appears to be a critical factor in radiosensitisation, with optimum enhancement occurring within short defined time periods in murine tumours (Siemann *et al.*, 1977). However, its importance is diminished by the concurrent use of nicotinamide (Chaplin *et al.*, 1993). Some of the previous studies of carbogen and oxygen breathing in man may have failed owing to the use of extended periods of PIBT (Keresteci and Rider, 1973; Rubin *et al.*, 1979). However, in one randomised controlled study in patients with carcinoma of the cervix breathing normobaric oxygen for 5–10 min before irradiation, both local recurrence and overall survival were improved by the addition of oxygen (Bergsjo and Kolstad, 1968).

On the assumption that both nicotinamide and carbogen breathing may improve radiosensitisation by their action on tumour hypoxia, there has been some attempt to date to measure the effects of the agents on tumour $pO_2$ distribution. Measurement of tumour $pO_2$ distribution was previously hampered by the lack of a reliable direct method. The computerised Eppendorf $pO_2$ histograph oxygen microelectrode system has now become established as a safe, reproducible

Correspondence: V Laurence
Received 12 July 1994; revised 23 January 1995; accepted 5 February 1995

and relatively non-invasive method for measuring $pO_2$ in accespible tumours and normal tissues (Kallinowski *et al.*, 1990; Vaupel *et al.*, 1991). This method has been used to study tumour $pO_2$ distribution changes in animal models following treatment with either nicotinamide or carbogen. Rises in $pO_2$ following nicotinamide treatment have been shown in only one study to date (Lee and Song, 1992), whereas others have shown no change (Kelleher and Vaupel, 1993). Significant rises have been shown following treatment with carbogen breathing (Horsman *et al.*, 1993*b*; Vitu-Loas *et al.*, 1993). In man, tumour $pO_2$ rises have been demonstrated during carbogen breathing in two studies to date (Falk *et al.*, 1992; Martin *et al.*, 1993) but $pO_2$ has not been measured following treatment with nicotinamide or the combination.

In the current study, we have measured changes in tumour $pO_2$ distribution in patients with advanced malignant disease following nicotinamide administration and during subsequent carbogen breathing. In addition, we have studied nicotinamide pharmacokinetics and the toxicity of the combined treatment.

### Materials and methods

### *Patient characteristics*

Twelve patients were entered into the study, of whom ten received the combination of nicotinamide and carbogen breathing before irradiation and had measurements of tumour $pO_2$ distribution performed. Two patients received nicotinamide alone and were eligible for toxicity and pharmacokinetic assessment. Table I shows the characteristics and tumour details of the treated patients. All had advanced recurrent or metastatic histologically proven malignancy requiring palliative irradiation, with accessible tumours of an adequate size to obtain a satisfactory number of $pO_2$ readings without tissue trauma. Tumours were not cystic, haemorrhagic or too firm for $pO_2$ measurements to be performed. Only one had been previously irradiated in the site used for $pO_2$ measurements (no. 5). All patients were of WHO performance status $\leqslant 2$ and had no serious cardiac or respiratory illness. All had haemoglobin values above 11 g $dl^{-1}$. Patients were defined as smokers if they had smoked at any time during the past 5 years. The protocol was approved by the local ethical committee and written informed consent was obtained from all patients.

### *Nicotinamide administration*

All patients received oral nicotinamide at a dose of 4–6 g as 500 mg tablets (Larkhall Natural Health, UK). It had been our intention to treat all patients with a standard dose of 6 g, but following unexpected acute toxicity in the second patient we modified our dose to 4 g until further experience had been gained. Tablets were taken 3–4 h after a light breakfast. No cigarettes, food or drink other than water were allowed after breakfast until completion of carbogen breathing.

### *Carbogen breathing*

Carbogen breathing was commenced as close to the estimated time of peak plasma nicotinamide concentration as possible. Published data suggested that this would be at around 90 min (Stratford *et al.*, 1992) and this time was used for the initial patients. However, as large individual variation was found, approximate nicotinamide concentrations were measured directly in the clinic in six patients (see Pharmacokinetic assessment below) and carbogen breathing was commenced at the time of individually estimated peak plasma nicotinamide concentrations. During carbogen breathing patients wore an air-tight scuba-diving face mask covering the nose and mouth (SOS Products) and were in control of holding the mask in place. The inhaled carbogen (95% oxygen, 5% carbon dioxide; from British Oxygen) was delivered to the face mask using a closed system with regulated flow rate of 4–5 l $min^{-1}$ through an anaesthetic ambubag and two-way valve (supplied by Intersurgical). Exhaled gas was removed using elephant tubing connected between the valve and a window to the outside. Patients were asked to breathe carbogen for as long as they were able to do so for up to 20 min. On completion of breathing carbogen, all patients were treated with palliative irradiation.

### *Pharmacokinetic assessment*

Serial 5 ml venous blood samples were collected from a heparinised 22 G cannula (Venflon, Viggo, UK) at regular time points after nicotinamide administration. Urine was also collected for 24 h. Plasma and urine nicotinamide concentrations were determined using a method of high-performance liquid chromatography (HPLC) analysis based on the procedure described by De Vries and Stratford (De Vries *et al.*, 1980; Stratford *et al.*, 1992). Briefly, 200 μl of plasma was added to 400 μl of acetonitrile containing 25 μg $ml^{-1}$ Ro-31-602 as an internal standard, and after vortexing the sample was centrifuged (9500 *g*, 5 min). A 50 μl aliquot of the supernatant was diluted with 150 μl of mobile phase and injected onto the HPLC system. Separations were achieved on a Nova-Pak $C_{18}$ (8 mm × 10 cm Radial Pak cartridge) with a mobile phase of 25% acetonitrile, and 75% of an aqueous solution of 2 mM sulphosuccinate 1 mM trimethylamine, 20 mM orthophosphoric acid and 5 mM sodium dihydrogen orthophosphate at a flow rate of 2 ml $min^{-1}$. Nicotinamide was detected by UV absorbance at 254 nm and quantified by peak area with reference to calibration standards using Waters Expert-Ease software V2.1.

Having found wide variability in the time of peak plasma nicotinamide concentration in initial patients, we subsequently used a rapid detection method in the clinic. Plasma nicotinamide concentration was estimated using an LKB UV spectrophotometer. This is an approximate method which does not distinguish between nicotinamide and its metabolites but nonetheless gives an indication of increasing and decreasing blood concentrations of detectable compounds. Briefly, plasma was obtained by centrifugation of 1 ml blood samples at 7500 *g* for 3 min. A 1 ml aliquot of

**Table I** Patient and tumour characteristics

| *Patient no.* | *Sex* | *Age (years)* | *Height (m)* | *Weight (kg)* | *Hb ($g\ dl^{-1}$)* | *Smoker in last 5 years* | *Tumour site* | *Tumour histology* | *Tumour volume ($cm^3$)* |
|---|---|---|---|---|---|---|---|---|---|
| 1 | M | 57 | - | 74 | 11.7 | Y | Skin | met AC caecum | 4 |
| 2 | M | 72 | 1.70 | 77 | 14.9 | N | Neck node | met SCC bladder | 8 |
| 3 | M | 68 | 1.77 | 79 | 14.4 | N | Chest wall | met SCC kidney | 11 |
| 4 | F | 58 | 1.58 | 73 | 12.1 | Y | Neck node | met SCLC | 23 |
| 5 | F | 64 | 1.58 | 75 | 13.9 | N | Groin node | met SCC cervix | 29 |
| 6 | M | 57 | 1.70 | 81 | 12.3 | Y | Suprasternal mass | met SCLC | 6 |
| 7 | F | 66 | 1.58 | 59 | 11.0 | N | Skin | met AC stomach | 57 |
| 8 | M | 71 | 1.75 | 78 | 12.5 | N | Groin node | met SCC bronchus | 28 |
| 9 | F | 68 | 1.60 | 52 | 11.1 | N | Breast | rec AC breast | 10 |
| 10 | M | 42 | 1.80 | 60 | 11.5 | Y | Neck node | met SCC bronchus | 64 |
| 11 | M | 86 | 1.71 | 56 | na | Y | Chest wall | met AC bronchus | 104 |
| 12 | F | 57 | 1.62 | 84 | 14.8 | N | Neck node | met AC breast | 113 |

met, metastatic; rec, recurrent; SCC, squamous cell carcinoma; SCLC, small cell lung cancer, AC, adenocarcinoma.

dried acetonitrile was added to 200 µl of plasma and the mixture was vortexed and then centrifuged for a further 3 min at 7500 *g*. The supernatant was transferred to a clean glass cuvette and measured at 260 nm. When sample absorbance levels showed a plateau or decline it was assumed that peak plasma nicotinamide concentration had been reached. The remainder of the initial 5 ml blood samples from these patients were stored on ice for subsequent HPLC analysis and comparison with spectrophotometer results.

*Assessment of toxicity*

Patients all had an initial full history and physical examination performed with particular attention to measurement of the tumour in which $pO_2$ measurements were to be performed. Resting pulse rate, blood pressure and arterial oxygen saturation (measured by pulse oximeter supplied by Omeda) were performed before and at 15 min intervals after nicotinamide administration and until completion of carbogen breathing. Any unexpected symptoms or change in vital signs were noted. On completion of the radiation treatment pulse rate and blood pressure were monitored every 4 h for 24 h. Patients were assessed for early toxicity by history and examination before discharge at 24 h and reassessed in the clinic 3–4 weeks after the procedure.

*Tumour oxygen measurements*

Tumour oxygen partial pressure $pO_2$ was measured using the computerised KIMOC 6650 Eppendorf $pO_2$ histograph as previously described in detail (Vaupel *et al.*, 1991). In brief, the polarographic electrode system was calibrated for 15 min in buffered sterile physiological saline with air and 100% nitrogen before and after each series of measurements. After initial calibration a 22 G intravenous cannula (Venflon) was inserted into or near the tumour following the subcutaneous injection of 2 ml of local anaesthetic (lignocaine 2% without adrenaline). Tumour temperature was measured using a tissue-implantable thermocouple microprobe (Physitemp) with BAT 10 data display system and was programmed into the histograph so that all $pO_2$ readings were corrected for temperature. The microelectrode probe was then inserted into the tumour through the cannula in order to protect the membrane of the cathode. The electrode was allowed to stabilise and the set of measurements then taken. The probe was programmed to move forwards in steps of 1 mm immediately followed by a step of 0.3 mm backwards in order to minimise tissue compression artefacts.

The track length and number of tracks taken were individually selected according to tumour size, and sets of readings were obtained from across the maximum diameter of the tumour. Patients remained supine in a warm and comfortable position during the procedure. Sets of measurements were performed before nicotinamide administration, at estimated peak plasma nicotinamide concentration (immediately before carbogen breathing) and then at 3–4 time points during carbogen breathing to obtain a time course. Each individual measurement was displayed on the histograph as it was taken and sets were then presented as frequency histograms.

*Evaluation of data and statistical analysis*

Sets of $pO_2$ measurements from each patient were transferred from the Eppendorf histograph to a Macintosh SE/30 and analysed using Statview software (Abacus Concepts) to obtain median values, inter-percentile ranges and percentages of values less than 10, 5 and 2.5 mmHg for individual patients. Statistical analysis was by the non-parametric Mann–Whitney *U*-test to assess the significance of any changes in $pO_2$ distribution after treatment with nicotinamide and during carbogen breathing. Intra- and inter-tumour heterogeneity were compared using analysis of variance and using the median $pO_2$ of each individual track as the parameter of oxygenation. Three time intervals were chosen based on data availabiality and compared using Snedecor's variance ratio test (Moroney, 1990). Pharmacokinetic data were analysed using Kaleidagraph software (Albeck) for the calculations of means, standard errors and correlation coefficients.

### Results

*Nicotinamide pharmacokinetics*

Pharmacokinetic data were analysed from 12 patients and are shown in Table II; four patients received a dose of 4 g, three received 5 g and five received 6 g. There was wide variation between patients in both peak plasma nicotinamide concentration and time to reach it. The mean peak plasma nicotinamide concentrations were 82, 115 and 150 µg ml$^{-1}$ for doses of 4, 5 and 6 g respectively, the overall mean ± 1(s.e.) being 115 (± 10) µg ml$^{-1}$. There was a linear relationship between peak plasma nicotinamide concentration and nicotinamide dose expressed either in g, mg kg$^{-1}$ or g m$^{-2}$ (correlation coefficients, $R = 0.73$, 0.68 and 0.73 respectively). Figure 1 shows the relationship between administered nicotinamide dose in grams and peak plasma nicotinamide concentration. In contrast, no relationship was found between administered nicotinamide dose and the time of peak plasma nicotinamide concentration, which had an inter-patient range of 0.7–4 h with a mean (± s.e.) of 2.4 (± 0.3) h.

We were particularly concerned to begin carbogen breathing as near to the time of peak plasma nicotinamide concentration as possible. Having found marked variation in this time in the initial patients we then used the spectrophotometric assay in six subsequent patients. We assumed that peak nicotinamide concentration had been reached when a plateau or fall in UV absorption was detected. At this stage a set of oxygen measurements was performed and then carbogen breathing commenced. A time lag between the true time of the peak and commencement of carbogen thus inevitably occurred. Figure 2 shows the comparative profiles of the percentages of peak plasma nicotinamide concentrations in the six patients as measured by the two different assays and their relationship to the time of carbogen breathing. It demonstrates that the rapid spectrophotometric assay gave a reasonable indication of the peak in all except one patient (no. 9). Of the five patients who breathed carbogen four (80%) had mean nicotinamide plasma concentrations (as measured by subsequent HPLC analysis) during gas breathing which were within 10% of the peak. In contrast, if a standard 90 min time to reach the peak had been assumed throughout the study, only 33% of the patients would have had nicotinamide plasma concentrations within 10% of the peak at the commencement of carbogen breathing (data not shown). Overall, the mean plasma concentration of nicotinamide during carbogen breathing was within 20% of peak plasma nicotinamide concentration in eight out of ten patients.

*Toxicity associated with nicotinamide*

After nicotinamide administration 9 out of 12 patients suffered from some toxicity (Table II). Diastolic blood pressure fell in three patients (> 15 mmHg diastolic), of whom two were asymptomatic and one had accompanying mild dizziness. Five patients noticed flushing, one accompanied by mild nausea, and three had mild headaches. One patient with a long-standing history of asymptomatic hypertension, concurrently receiving bendrofluazide medication, suffered significant toxicity at the time of peak nicotinamide concentration, with profound hypotension resulting in a transient cardiorespiratory arrest. He quickly regained consciousness but remained relatively bradycardic and hypotensive for several hours. He recovered completely with no further sequelae.

**Table II** Summary of nicotinamide pharmacokinetics

| Patient no. | Surface area[a] ($m^2$) | Dose (g) | Dose ($mg\ kg^{-1}$) | Dose ($g\ m^{-2}$) | $C_{peak}$[b] ($\mu g\ ml^{-1}$) | $T_{peak}$[c] (h) | $t_{1/2\beta}$[d] (h) | $AUC_{0\rightarrow\infty}$[e] ($\mu g\ ml^{-1}\ h$) | NCT concentration during carbogen breathing ($\mu g\ ml^{-1}$) | Side-effects |
|---|---|---|---|---|---|---|---|---|---|---|
| 1 | – | 6 | 81 | – | 138 | 0.7 | 6.5 | 1400 | 118 | Vertigo, hypotension |
| 2 | 1.9 | 6 | 78 | 3.2 | 136 | 1.7 | 9.5 | 2400 | – | Nausea, vomiting, hypotension, cardiorespiratory arrest |
| 3 | 1.9 | 4 | 51 | 2.1 | 55 | 3.6 | 3.5 | 380 | 35 | – |
| 4 | 1.7 | 4 | 55 | 2.3 | 78 | 1.5 | 1.9 | 760 | 63 | – |
| 5 | 1.8 | 4 | 53 | 2.3 | 102 | 1.0 | 6.6 | 930 | 91 | Flushing, headache |
| 6 | 1.9 | 5 | 62 | 2.6 | 151 | 2.0 | 6.1 | 1900 | 145 | – |
| 7 | 1.6 | 4 | 68 | 2.5 | 94 | 3.6 | 2.7 | 990 | 91 | – |
| 8 | 1.9 | 5 | 65 | 2.6 | 89 | 3.1 | 2.5 | 1200 | 82 | Flushing, headache, hypotension |
| 9 | 1.5 | 5 | 96 | 3.3 | 105 | 4.0 | 3.6 | 1200 | 81 | Headache, hypotension |
| 10 | 1.8 | 6 | 100 | 3.4 | 177 | 2.0 | 7.1 | 2300 | 165 | Flushing |
| 11 | 1.6 | 6 | 108 | 3.6 | 146 | 1.5 | 10.7 | 2400 | – | Flushing |
| 12 | 1.8 | 6 | 71 | 3.2 | 103 | 4.0 | 7.4 | 1800 | 97 | Flushing, nausea |

[a]Surface area, estimated from a normagram; [b]$C_{peak}$, peak nicotinamide plasma concentration; [c]$T_{peak}$, time of peak nicotinamide plasma concentration; [d]$t_{1/2\beta}$, nicotinamide plasma elimination half-life; [e]$AUC_{0\rightarrow\infty}$, area under curve.

**Figure 1** Peak plasma concentration of nicotinamide (NCT) as a function of dose in g.

### *Carbogen breathing*

Of the 12 patients in the study, two did not breathe carbogen. The patient in whom major toxicity occurred was obviously unable to complete the study, and patient no. 11 was able to tolerate nicotinamide but judged to be too frail to breathe carbogen. Carbogen breathing was well tolerated by all remaining patients. It was performed for between 15 and 20 min in all except one (no 1), who was only able to complete 10 min of gas breathing. One patient removed his mask for 4 min after 13 min (no. 6) and then recommenced breathing again for a further 3 min. During carbogen breathing the mean time to reach 100% arterial oxygen saturation was 2.35 min (range 0.5–6 min).

### *Toxicity associated with combined treatment*

Following radiation treatment all patients were assessed at 24 h and at 3–4 weeks. No obvious increase in normal tissue toxicity was noted.

### *Tumour oxygenation*

Sets of tumour $pO_2$ measurements were obtained in ten patients. Between 30 and 150 measurements were performed per set of readings using typically 3–4 tracks. Table III shows the number of readings per set and the parameters of tumour oxygenation in terms of the median $pO_2$ value in mmHg and the percentage of values $<5$ mmHg obtained during each set of readings. We have chosen to present the value of $\% < 5$ mmHg to represent hypoxia as only one patient had $>1\%$ of readings of $<2.5$ mmHg before treatment (Hall, 1987). Table IV shows the estimates of variance, using median $pO_2$ as the parameter of oxygenation, before treatment, at presumed peak nicotinamide concentration and during carbogen breathing. At all three time points there was greater variation of oxygenation between tumours than within tumours, confirming that despite wide inter-percentile ranges per set an adequate number of readings had been taken to exclude any bias (Nordsmark *et al.*, 1994).

Figure 3 shows the changes in median $pO_2$ and the interpercentile ranges (10–90%) for each set of measurements performed in all patients. Following nicotinamide only two patients had significant rises in median tumour $pO_2$ ($P < 0.05$) compared with pretreatment. During carbogen breathing, and comparing $pO_2$ distributions with those obtained after nicotinamide, nine out of ten patients had highly significant rises in median tumour $pO_2$ ($P < 0.0001$). These rises occurred within 10 min of commencing carbogen in all except one patient (no. 4) and continued

**Figure 2** Percentages of peak plasma concentration of nicotinamide (NCT), as measured by (○) HPLC and (●) spectrophotometer assay system, as a function of time after oral administration of NCT in six patients and their relationship to the commencement of carbogen breathing (↑).

**Figure 3** Median $P_{O_2}$ values and 10–90% inter-percentile ranges in ten patients before ingestion of nicotinamide (pre), at presumed peak plasma nicotinamide concentration (post-NCT) and at times 0–5, 5–10, 10–15 and > 15 min after commencement of carbogen breathing.

to rise significantly or remain in plateau in all except two (nos. 10 and 12). Of the six patients with pretreatment values of less than 5 mmHg, these had been completely abolished in three and were reduced in two during carbogen breathing. Patient no. 5 was the only patient who had previously been irradiated in the measured site and was also the only patient to show no significant rise in tumour median $pO_2$.

### Discussion

The aim of this study was to investigate whether combining treatment with nicotinamide and carbogen breathing altered $pO_2$ distribution in accessible tumours of patients with advanced malignant disease. In addition, data were obtained on changes in $pO_2$ after nicotinamide alone, nicotinamide pharmacokinetics and toxicity of the combined treatment.

The nicotinamide pharmacokinetic data confirmed and extended those for man already in the literature. Following doses of 4–6 g in tablet formation (51–108 mg $kg^{-1}$) the magnitude of peak concentration and plasma clearance were consistent with previous findings on normal volunteers (Stratford *et al.*, 1992; Horsman *et al.*, 1993*a*) and were both dose dependent. The time to reach peak plasma concentration was widely variable and was independent of dose, as in Stratford's study using the same formulation, but unlike

**Table III** Summary of oxygen distribution parameters in individual patients

| Patient no. | Pretreatment Median | Pretreatment %<5 | Pretreatment n | Post-nicotinamide Median | Post-nicotinamide %<5 | Post-nicotinamide n | 0–5 min Median | 0–5 min %<5 | 0–5 min n | 5–10 min Median | 5–10 min %<5 | 5–10 min n | 10–15 min Median | 10–15 min %<5 | 10–15 min n | 15+ min Median | 15+ min %<5 | 15+ min n |
|---|---|---|---|---|---|---|---|---|---|---|---|---|---|---|---|---|---|---|
| | | | | | | | $pO_2$ measurements taken during carbogen breathing | | | | | | | | | | | |
| 1 | 2 | 60 | 60 | – | – | – | 1 | 88 | 132 | 15 | 38 | 68 | – | – | – | – | – | – |
| 3 | 38 | 0 | 150 | 39 | 0 | 96 | 51 | 0 | 108 | 69 | 0 | 108 | 60 | 0 | 72 | 73 | 0 | 72 |
| 4 | 10 | 0 | 105 | 11 | 0 | 84 | 8 | 0 | 84 | 19 | 0 | 105 | 12 | 0 | 84 | 43 | 2 | 63 |
| 5 | 11 | 0 | 129 | 7 | 27 | 55 | 17 | 31 | 102 | 13 | 35 | 84 | 4 | 52 | 101 | 12 | 36 | 99 |
| 6 | 15 | 24 | 63 | 30 | 36 | 93 | 38 | 0 | 79 | 54 | 0 | 42 | 44 | 0 | 53 | – | – | – |
| 7 | 30 | 14 | 96 | 6 | 44 | 32 | 19 | 32 | 107 | 85 | 8 | 63 | 137 | 0 | 30 | 123 | 8 | 65 |
| 8 | 5 | 50 | 84 | – | – | – | 31 | 37 | 49 | 45 | 19 | 74 | 62 | 9 | 56 | – | – | – |
| 9 | 61 | 18 | 116 | 26 | 21 | 94 | 56 | 6 | 84 | 63 | 0 | 50 | – | – | – | 81 | 0 | 42 |
| 10 | 43 | 0 | 89 | 64 | 0 | 56 | 103 | 0 | 55 | 103 | 0 | 60 | 65 | 0 | 54 | – | – | – |
| 12 | 52 | 0 | 125 | 48 | 0 | 63 | 87 | 0 | 63 | 88 | 0 | 42 | 76 | 6 | 63 | 59 | 19 | 42 |

Median, median $Po_2$ in mmHg; %<5, percentage of $Po_2$ readings less than 5 mmHg; N, number of tracks.

Horsman's results using a capsule formulation, after which the time to reach peak concentration occurred predictably within 45 min of ingestion. There is clear evidence in an animal system that maximum radiosensitisation by nicotinamide occurs at the time of peak plasma concentration and surprisingly appears to be independent of the magnitude of the peak (Horsman *et al.*, 1993*a*). The efforts made in this study to deliver carbogen at the time of peak nicotinamide concentration were largely successful. The spectrophotometric assay was cumbersome but allowed reasonable assessment of the time of the peak. Nonetheless, a capsule or rapid release formulation with a more predictable time of peak concentration would clearly be an advantage.

There has been some debate about the required dose of nicotinamide in humans which might achieve significant radiosensitisation without toxicity (Brown, 1992). Initial studies suggested that optimum sensitisation could only be achieved in murine tumours with intraperitoneal (i.p.) doses of over 500 mg $kg^{-1}$ which were thought to translate into a dose in man which would be unacceptable in terms of toxicity (Horsman *et al.*, 1987). In a recent study of the comparative pharmacokinetics of nicotinamide in mouse and man, oral doses of 6 g in man gave rise to similar peak plasma levels as 171 mg $kg^{-1}$ i.p. nicotinamide in mice (Horsman *et al.*, 1993*a*), a dose which produced maximal enhancement of radiation damage in the mouse tumours provided that the radiation was delivered at the time of peak drug level. Thus assuming that a similar mechanism occurs in man, a 6 g dose should be adequate to ensure enhancement of radiosensitisation provided that the radiation can be given at the time of peak plasma drug levels. This is a substantially smaller dose than was originally thought to be required.

Although 6 g is thought to be a safe dose in man, there still remain questions about potential toxicity. Neither study of nicotinamide pharmacokinetics in man reported major toxicity in healthy volunteers, although single instances of migraine and nausea and vomiting were reported (Stratford *et al.*, 1992; Horsman *et al.*, 1993*a*). Nicotinamide has been extensively and safely used in repeat dosage of up to 6 g in the treatment of a number of benign conditions (Zackheim, 1981), but in the current study of patients with malignant disease most complained of minor side-effects. The hypotensive episode occurring at peak plasma nicotinamide concentration in one patient in the study is a cause for greater concern. It is likely that this event was related to his concurrent diagnosis of hypertension and antihypertensive medication, but caution still needs to be maintained until further experience is gained of this drug in patients with malignant disease.

The precise mode of action of nicotinamide as a radiosensitiser in animal tumour models remains unclear. It is thought to be a result of improved tumour oxygenation, as shown indirectly by a reduction in [$^{14}C$]misonidazole binding (Horsman *et al.*, 1988) and specifically a decrease in acute hypoxia as demonstrated using the histological mismatch technique (Chaplin *et al.*, 1990). The observed effects of nicotinamide on blood perfusion and tumour $pO_2$ as measured directly by microelectrodes have not been consistent. Data from one study demonstrated increases in tumour oxygen distribution in the FSaII mouse tumour model following treatment with 500 mg $kg^{-1}$ i.p. nicotinamide in association with increased blood perfusion and increased radiosensitivity (Lee and Song, 1992). In another study using a different tumour model, significant increases in hypoxic values (<10 mmHg) were found despite an improvement in tumour blood flow (Kelleher and Vaupel, 1993).

In contrast, carbogen breathing has been found to increase median $pO_2$ and to decrease hypoxic values in several animal studies (Horsman *et al.*, 1993*b*; Vitu-Loas *et al.*, 1993). Of particular importance has been our previous demonstration in RIF-1 tumours grown in C3H mice that, while nicotinamide produced no significant rise in tumour $pO_2$ distribution, carbogen breathing gave rise to large increases in median $pO_2$. Maximum increases occurred when nicotinamide and carbogen breathing were combined (Honess

**Table IV** Estimates of variance, using median $Po_2$ as oxygenation parameter

| | *Sum of squares* | *Degrees of freedom* | *Variance estimate* | *F* | P-*value* |
|---|---|---|---|---|---|
| Pretreatment ($n$ = 10) | | | | | |
| Between patients | 11385 | 9 | 1265 | | |
| Between tracks in a patient | 14828 | 29 | 511 | 2.47 | $0.01 < P < 0.05$ |
| Post-NCT ($n$ = 8) | | | | | |
| Between patients | 7585 | 7 | 1084 | | |
| Between tracks in a patient | 3478 | 15 | 232 | 4.7 | $0.005 < P < 0.01$ |
| During carbogen breathing at 5–10 min ($n$ = 10) | | | | | |
| Between patients | 26071 | 9 | 2897 | 9.3 | $P < 0.001$ |
| Between tracks in a patient | 5625 | 18 | 313 | | |

$F$ = Snedecor's variance ratio. $P$-value obtained from $F$-distrubiton tables, using the tabulated degrees of freedom.

*et al.*, 1994). These observations are consistent with the findings of maximum enhancement of radiosensitisation occurring after combining the agents (Kjellen *et al.*, 1991).

In man there have been no other published studies of the measurement of tumour $pO_2$ distribution following combined treatment with nicotinamide and carbogen breathing. The findings of greater between- than within-tumour heterogeneity at three time points support the impression of wide individual variation between tumours and make pooling of individual patient data irrelevant. Each individual was therefore assessed separately, and nine out of ten showed significant rises in tumour oxygen distribution. As in our previous study of carbogen breathing alone (Falk *et al.*, 1992), we found a wide range of change in median $pO_2$ with elimination of some but not all values of $pO_2$ below 5 mmHg. The rises in median $pO_2$ tended to be of greater magnitude than in the previous study and were sustained until 15 min and over in the majority of our patients, whereas in the previous study a fall in median tumour $pO_2$ occurred in all patients between 12 and 18 min.

Our current results suggest that the combination of nicotinamide and carbogen breathing may give rise to greater improvements in tumour hypoxia than carbogen breathing alone, and in particular may result in a sustained rise rather than a rise followed by a progressive fall. These results are supported by experimental animal data. Thus Chaplin *et al.* (1993) have shown that the influence of the length of the pre-irradiation breathing time of carbogen on radiosensitivity was diminished by the concurrent use of nicotinamide, following which hypoxia was apparently abolished. Our findings, overall, support the results in experimental tumour models of a greater reduction in tumour hypoxia after the use of the combined agents compared with either agent alone. In addition, the combination appears to be effective over a longer time period than carbogen alone, which in the clinic would ensure less risk of missing the time of presumed tumour radiosensitisation.

In conclusion, we have treated ten patients with advanced malignant disease with the combination of oral nicotinamide and carbogen breathing before palliative radiotherapy and a further two patients received nicotinamide alone. Side-effects were generally mild except for one patient who suffered from a major toxic event. Despite being unable to demonstrate marked changes in tumour oxygen distribution close to peak plasma nicotinamide concentration and therefore at assumed optimum time for radiosensitisation, large and highly significant rises in median tumour $pO_2$ were demonstrated during carbogen breathing which were sustained for at least 15 min. Although these changes in $pO_2$ cannot be shown to be occurring in the milieu of the radioresistant hypoxic cells, our results are encouraging and should stimulate further clinical studies of this potentially highly effective combination of agents in order to establish whether the impressive therapeutic gains seen in animal models will be repeatable in man.

**Acknowledgements**
We would like to thank Dr Davina Honess for helpful discussions and criticisms of this work.

**References**

BERGSJØ P AND KOLSTAD P. (1968). Clinical trial with atmospheric oxygen breathing during radiotherapy of cancer of the cervix. *Scand. J. Clin. Lab. Invest. (Suppl)*, **106**, 167–171.

BROWN JM. (1992). Carbogen and nicotinamide: expectations too high? *Radiother. Oncol.*, **24**, 75–76.

CHAPLIN DJ, HORSMAN MR AND TROTTER MJ. (1990). Effect of nicotinamide on the microregional heterogeneity of oxygen delivery within a murine tumour. *J. Natl Cancer Inst.*, **82**, 672–676.

CHAPLIN DJ, HORSMAN MR AND SIEMANN DW. (1993). Further evaluation of nicotinamide and carbogen as a strategy to reoxygenate hypoxic cells *in vivo*: importance of nicotinamide dose and preirradiation breathing time. *Br. J. Cancer*, **68**, 269–273.

COLEMAN CN. (1988). Hypoxia in tumours: a paradigm for the approach to biochemical and physiological heterogeneity (review). *J. Natl Cancer Inst.*, **80**, 310–317.

DE VREIS JX, GUNTHERT W AND DING R. (1980). Determination of nicotinamide in human plasma and urine by ion-pair reversed phase high-performance liquid chromatography. *J. Chromatogr.*, **221**, 161–165.

DISCHE. (1985). Chemical sensitisers for hypoxic cells: a decade of experience in clinical radiotherapy. *Radiother. Oncol.*, **3**, 97–115.

DISCHE S. (1991). Hypoxia and local tumour control. Part 2. *Radiother. Oncol.*, Suppl. **20**, 9–11.

FALK SJ, WARD R AND BLEEHEN NM. (1992). The influence of carbogen breathing on tumour tissue oxygenation in man evaluated by computerised $Po_2$ histography. *Br. J. Cancer*, **66**, 919–924.

HALL EJ. (1987). The oxygen effect and reoxygenation. In *Radiobiology for the Radiologist*, pp 139–160. Harper & Row: Philadelphia.

HENK JM. (1981). Does hyperbaric oxygen have a future in radiation therapy? *Int. J. Radiat. Oncol. Biol. Phys.*, **7**, 1125–1128.

HONESS D, LAURENCE V, WARD R, SHAW J AND BLEEHEN N. (1995). The effects of nicotinamide and carbogen, individually or in combination, on RIF-1 tumour oxygenation. In *Tumour oxygenation*, Vaupel P, Kelleher DK and Günderoth M. (eds). pp. 137–144. Gustav Fischer: Stuttgart.

HORSMAN MR, CHAPLIN DJ AND BROWN JM. (1987). Radiosensitisation by nicotinamide *in vivo*: a greater enhancement of tumour damage compared to that of normal tissues. *Radiat. Res.*, **109**, 479–489.

HORSMAN MR, BROWN JM, HIRST VK, LEMMON MJ, WOOD PJ, DUNPHY EP AND OVERGAARD J. (1988). Mechanism of action of the selective tumour radiosensitizer nicotinamide. *Int. J. Radiat. Oncol. Biol. Phys.*, **15**, 658–690.

HORSMAN MR, CHAPLIN DJ AND BROWN JM. (1989). Tumour radiosensitization by nicotinamide: a result of improved perfusion and oxygenation. *Radiat. Res.*, **118**, 139–150.

HORSMAN MR, HØYER M, HONESS DJ, DENNIS IF AND OVERGAARD J. (1993*a*). Nicotinamide pharmacokinetics in humans and mice: a comparative assessment and the implications for radiotherapy. *Radiother. Oncol.*, **27**, 131–139.

HORSMAN MA, KHALIL AA, NORDSMARK M, GRAU C AND OVERGAARD J. (1993*b*). Relationship between radiobiological hypoxia and direct estimates of tumour oxygenation in a mouse tumour model. *Radiother. Oncol.*, **28**, 69–71.

KALLINOWSKI F, ZANDER R, HÖCKEL M AND VAUPEL P. (1990). Tumour tissue oxygenation as evaluated by computerised $pO_2$ histography. *Int. J. Radiat. Oncol. Biol. Phys.*, **19**, 953–961.

KELLEHER DK AND VAUPEL PW. (1993). Nicotinamide exerts different acute effects on microcirculatory function and tissue oxygenation in rat tumours. *Int. J. Radiat. Oncol. Biol. Phys.*, **26**, 95–102.

KERESTECI AG AND RIDER WD. (1973). Use of orthobaric oxygen in the radiotherapy of bladder tumours. *Can. J. Surg.*, **16**, 127–129.

KJELLEN E, JOINER MC, COLLIER JM, JOHNS H AND ROJAS A. (1991). A therapeutic benefit from combining normobaric carbogen or oxygen with nicotinamide in fractionated X-ray treatments. *Radiother. Oncol.*, **22**, 81–91.

KRUUV JA, INCH WR AND McCREDIE JA. (1967). Blood flow and oxygenation of tumours in mice. *Cancer*, **20**, 51–59.

LEE I AND SONG CW. (1992). The oxygenation of murine tumour isografts and human tumour xenografts by nicotinamide. *Radiat. Res.*, **130**, 65–71.

MARTIN L, LARTIGAU E, WEEGER P, LE RIDANT AM, LUSINCHI A, WIBAULT P, ESCHWEGE F, LUBOINSKI B AND GUICHARD M. (1993). Changes in the oxygenation of head and neck tumours during carbogen breathing. *Radiother. Oncol.*, **27**, 123–130.

MORONEY MJ. (1990). The analysis of variation and co-variation. In *Facts from Figures*, pp. 371–457. Penguin Books: London.

NORDSMARK M, BENTZEN SM AND OVERGAARD J. (1994). Measurement of human tumour oxygenation status by a polarographic needle electrode. *Acta Oncol.*, **33**, 383–389.

OVERGAARD J. (1992). Importance of tissue hypoxia in radiotherapy. A meta-analysis of controlled clinical trials (abstract). *Radiother. Oncol.*, **24** (Suppl.), 247.

ROJAS A. (1991). Radiosensitisation with normobaric oxygen and carbogen. *Radiother. Oncol.*, **20** (Suppl.), 65–70.

ROJAS A. (1992). ARCON: accelerated radiotherapy with carbogen and nicotinamide. *Br. J. Radiol.*, **24**, 174–178.

RUBIN P, HANLEY J, KEYS HM. (1979). Carbogen breathing during radiation therapy. *Int. J. Radiat. Oncol. Biol. Phys.*, **5**, 1963–1970.

SIEMANN DW, HILL RP AND BUSH RS. (1977). The importance of the pre-irradiation breathing times of oxygen and carbogen (5% $CO_2$: 95% $O_2$) on the *in vivo* radiation response of a murine sarcoma. *Int. J. Radiat. Oncol. Biol. Phys.*, **2**, 903–911.

SIMON JM, LARTIGAU E AND GUICHARD M. (1993). Nicotinamide and carbogen: major effect on the radiosensitivity of EMT6 and HRT18 tumours. *Radiother. Oncol.*, **28**, 203–207.

STRATFORD MRL, ROJAS A, HALL DW, DENNIS MF, DISCHE S, JOINER MC AND HODGKISS RJ. (1992). Pharmacokinetics of nicotinamide and its effect on blood pressure, pulse and body temperature in normal human volunteers. *Radiother. Oncol.*, **25**, 37–42.

VAUPEL P, SCHLENGER K, KNOOP C AND HÖCKEL M. (1991). Oxygenation of human tumours: evaluation of tissue oxygen distribution in breast cancers by computerised $O_2$ tension measurements. *Cancer Res.*, **51**, 3316–3322.

VITU-LOAS L, THOMAS C, CHAVAUDRA N AND GUICHARD M. (1993). Radiosensitivity, blood perfusion and tumour oxygenation after perflubron emulsion injection. *Radiother. Oncol.*, **27**, 149–155.

ZACKHEIM R, (1981). Reactions to niacinamide (letter). *J. Am. Acad. Dermatol.*, **4**, 736–737.